# GCN2 in proteostasis: structural logic, signalling networks and disease

**DOI:** 10.1111/febs.70480

**Published:** 2026-03-04

**Authors:** JiaYi Zhu, Stefan J. Marciniak

**Affiliations:** ^1^ Cambridge Institute for Medical Research (CIMR), University of Cambridge UK; ^2^ Department of Medicine University of Cambridge UK; ^3^ Royal Papworth Hospital, NHS Foundation Trust UK

**Keywords:** amino acid sensing, GCN2, integrated stress response, proteostasis, translational control

## Abstract

Proteostasis is the finely tuned balance of protein synthesis, folding and degradation essential for cellular health. When this equilibrium is disrupted, misfolded proteins accumulate, triggering adaptive stress responses such as the unfolded protein response and the integrated stress response (ISR). Central to the ISR is the kinase GCN2, a sensor of amino acid deprivation and ribosomal stress. Upon activation, GCN2 phosphorylates eIF2α, dampening global translation while selectively enhancing the synthesis of the stress‐responsive transcription factors ATF4 and CHOP. ATF4 orchestrates a broad transcriptional programme that supports amino acid metabolism, redox homeostasis, autophagy and proteasomal degradation, which are key processes for restoring proteostasis. Beyond its canonical role, GCN2 interfaces with other regulatory networks modulating mTORC1 to promote autophagic clearance of damaged proteins and organelles, facilitating stress granule formation, and integrating signals from oxidative and endoplasmic reticulum stress to rebalance the proteome. Dysregulated GCN2 activity has been implicated in diverse pathologies including neurodegeneration, cancer and pulmonary vascular disease, positioning it as a promising therapeutic target. In this review, we explore how GCN2 links nutrient sensing to translational control and metabolic adaptation, and how its central role in proteostasis may inform new strategies for treating diseases driven by protein misfolding and stress pathway imbalance.

## Graphical abstract

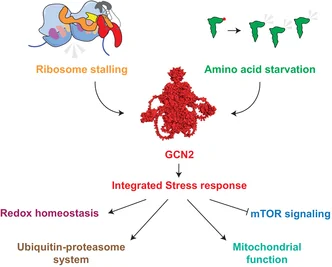

**Abbreviations**
ATF3: activating transcription factor 3
ATF4: activating transcription factor 4
ATP: adenosine triphosphate.
CAT1: cationic amino acid transporter 1
CHOP: C/EBP‐homologous protein
CReP: constitutive repressor of eIF2α phosphorylation (PPP1R15B)
CTD: C‐terminal domain
DFG: Asp‐Phe‐Gly motif (kinase activation loop)
DR5: death receptor 5
eEF1A: eukaryotic elongation factor 1A
eEF2: eukaryotic elongation factor 2
eIF: eukaryotic initiation factor
eIF2: eukaryotic initiation factor 2
eIF2B: guanine nucleotide exchange factor for eIF2
eIF2α: eukaryotic initiation factor 2 alpha subunit
ER: endoplasmic reticulum
FBXO22: F‐box only protein 22
GADD34: growth arrest and DNA damage‐inducible protein 34 (PPP1R15A)
GTP: guanosine triphosphate
HISRS(−like): histidyl‐tRNA synthetase‐like domain
HO‐1: haem oxygenase‐1
IDO1: indoleamine‐2,3‐dioxygenase 1
ISR: integrated stress response
KD: kinase domain
LC3B: microtubule‐associated protein 1 light chain 3 beta
MAPK: mitogen‐activated protein kinase
MMC: mitomycin‐C
mRNA: messenger RNA
mTORC1: mechanistic target of rapamycin complex 1
PAH: pulmonary arterial hypertension
PCH: pulmonary capillary haemangiomatosis
PERK: PKR‐like ER kinase
PKR: protein kinase R
PP1: protein phosphatase 1
P‐stalk: ribosomal P‐protein stalk
PVOD: pulmonary veno‐occlusive disease
RQC: ribosome quality control
RWD: RING‐finger proteins, WD‐repeat proteins and DEAD‐like helicases domain
SMAD1/5/8: mothers‐against‐decapentaplegic homologues 1, 5 and 8
tRNA: transfer RNA
uL10: large‐subunit ribosomal protein L10
uORF: upstream open reading frame
UPS: ubiquitin–proteasome system
UV: ultraviolet

## Introduction

### GCN2: a central regulator of proteostasis and the integrated stress response

#### Proteostasis and translational control at the initiation step

Proteostasis encompasses diverse processes that maintain the integrity of the cellular proteome, ensuring that proteins are accurately synthesized, folded, modified, trafficked and ultimately degraded [1, 2, 3]. This balance is continuously challenged, and cells rely on tightly regulated translational and stress response pathways to preserve homeostasis.

**Fig. 1:**
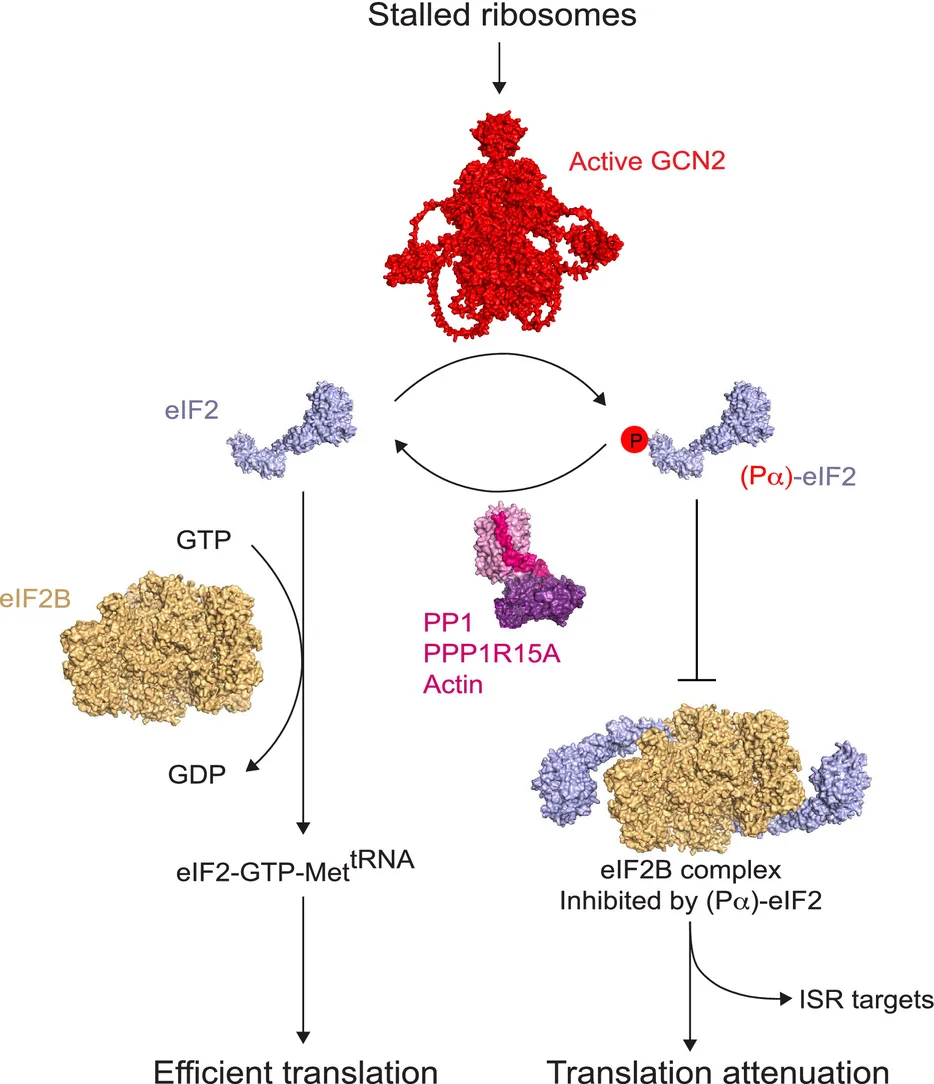
GCN2 activates the integrated stress response. Under basal conditions, the eIF2 complex is recharged with GTP by eIF2B, enabling the delivery of methionyl‐tRNA to the ribosome during translation initiation (PDB 6I3M [202]). When ribosomes stall, GCN2 becomes activated and phosphorylates the α‐subunit of eIF2, generating (Pα)‐eIF2. This phosphorylated form binds tightly to eIF2B and inhibits its guanine‐nucleotide exchange activity, leading to a global reduction in translation and a selective increase in the translation of integrated stress response (ISR) targets, including ATF4, CHOP and PPP1R15A. Ultimately, PPP1R15A, in complex with PP1 and actin, dephosphorylates (Pα)‐eIF2 to restore normal translation (PDB 7NZM [203]).

Translation initiation is a major control point in protein synthesis. Here, eukaryotic initiation factors (eIFs) recruit mRNA to the ribosome and position it at the start codon [4, 5, 6, 7]. A central regulatory mechanism, conserved across eukaryotes, involves phosphorylation of the eIF2α subunit [8, 9]. Under basal conditions, the eIF2 complex delivers initiator methionyl‐tRNA to the start codon [9, 10], hydrolysing GTP in the process [11, 12]. eIF2B then exchanges GDP for GTP, enabling further rounds of initiation [13, 14]. During stress, phosphorylated eIF2 binds tightly to eIF2B, inhibiting nucleotide exchange [13, 15, 16]. The resulting reduction in eIF2‐GTP‐ tRNAiMet ternary complex availability attenuates global cap‐dependent translation [13, 16, 17] (Fig. 1).

#### GCN2–eIF2α–ATF4 axis in proteostasis and stress adaptation

GCN2 (General Control Nonderepressible 2) is a conserved eIF2α kinase that responds to amino acid deprivation and ribosomal stress by phosphorylating serine 51 of eIF2α [18, 19]. Whereas unicellular eukaryotes typically encode a single eIF2α kinase, metazoans have diversified this system: Multiple kinases share a conserved catalytic domain but possess distinct stress‐sensing modules, enabling them to detect a broad range of perturbations [20, 21, 22]. Because these kinases converge on eIF2α phosphorylation to elicit a common adaptive programme, their collective output is termed the integrated stress response (ISR) [19, 23, 24, 25, 26].

A hallmark of the ISR is the selective translation of specific mRNAs despite global translational repression. This phenomenon was first characterised in yeast, where gcn4 translation increases during amino acid starvation [27, 28, 29]. In mammals, ATF4 serves as the functional orthologue: its mRNA contains upstream open reading frames (uORFs) that allow translation to be enhanced when ternary complex levels fall [29, 30, 31, 32, 33]. Under non‐stress conditions, ribosomes translate uORF1 and uORF2, rapidly reacquire ternary complex, and initiate at the inhibitory uORF3, thereby preventing ATF4 expression [30, 32]. During stress, reduced ternary complex availability increases the likelihood that ribosomes bypass uORF3 and initiate at the ATF4 coding sequence [29, 33]. ATF4 then induces genes involved in amino acid transport and biosynthesis [19, 30, 34] and CHOP, a transcription factor that amplifies the ISR programme [35, 36, 37, 38].

Termination of the ISR requires dephosphorylation of eIF2α. Vertebrate cells express two eIF2α phosphatases that counteract GCN2 and other eIF2α kinases [39, 40, 41]. Under basal conditions, a holophosphatase composed of PP1, the regulatory subunit PPP1R15B (CReP), and G‐actin maintains low levels of eIF2α phosphorylation [40, 42]. Although yeast and nematodes lack a dedicated eIF2α phosphatase, a PPP1R15 orthologue is present in *Drosophila* suggesting an ancient origin [43]. During stress, mammalian cells induce PPP1R15A (GADD34) in an ATF4 and CHOP dependent manner [39, 40, 41]. This forms a negative‐feedback loop that restores eIF2α to its unphosphorylated state once homeostasis can be re‐established [39, 40, 44, 45] (Fig. 1).

#### Structural basis of GCN2 stress sensing and activation

**Fig. 2:**
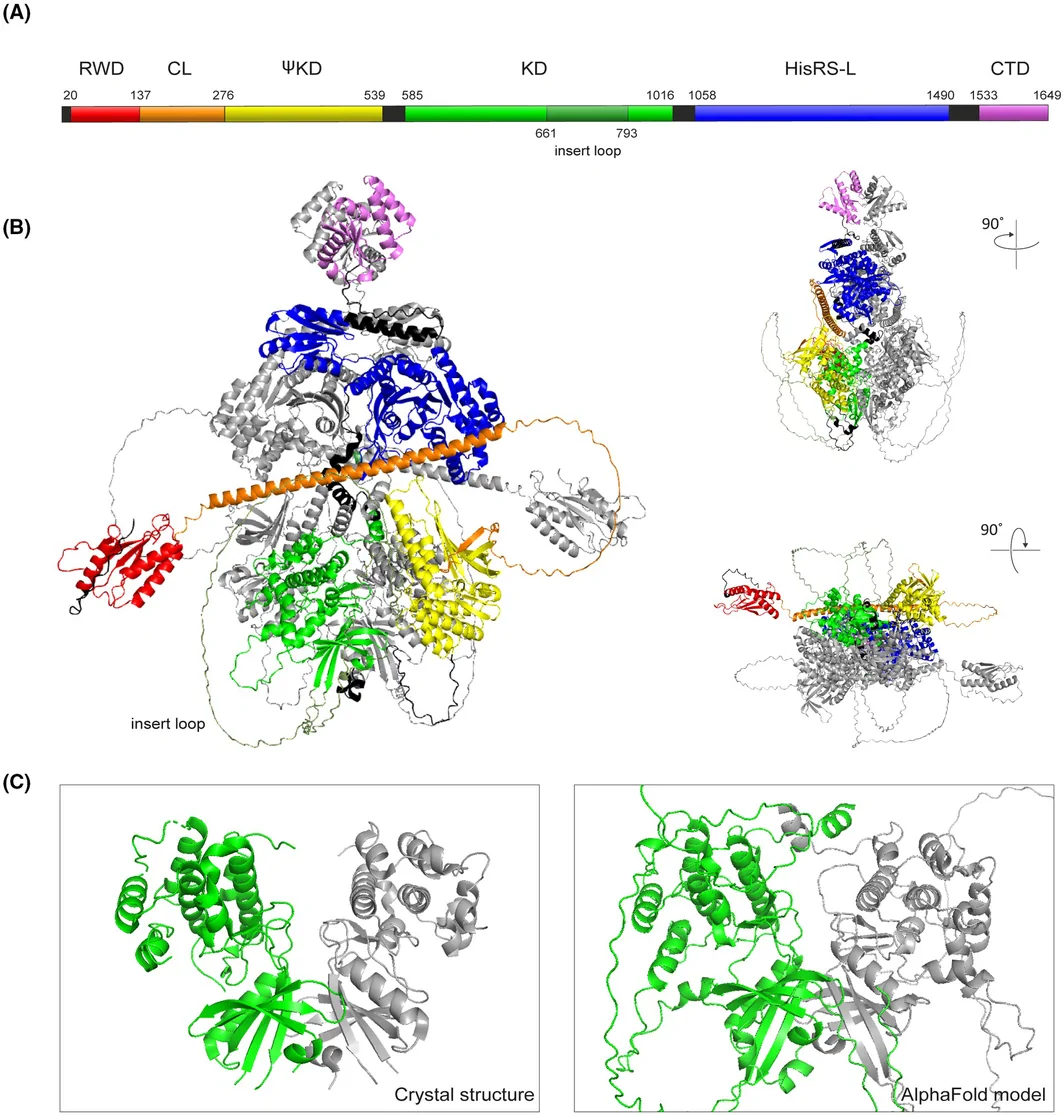
GCN2 structure. (A) Domain structure of GCN2: RWD (RING‐finger proteins, WD repeat‐containing proteins) and the yeast DEAD‐like helicases) domain, CL (charged linker), ѰKD (pseudokinase domain), KD (kinase domain), HisRS‐L (histidyl‐tRNA synthetase‐like domain) and a CTD (C‐terminal domain). The low complexity insert loop between strands β4 and β5 of the KD is indicated. After [200]. (B) alphafold model of dimeric human GCN2 (NP_001013725.2) with domains of one protomer coloured according to (A). The second protomer remains grey. Structures were displayed using pymol 3.0.2 (Schrödinger, LLC). (C) Comparison of crystal structure (PDB 7QWK [50]) of human GCN2 KD (left, bound compound 2 not shown) and alphafold generated model. Structures were displayed using pymol 3.0.2 (Schrödinger, LLC).

Human GCN2 is a multidomain kinase that operates as a cytosolic homodimer [46, 47]. Although long recognised as a central regulator of amino acid stress, only in recent years has its architecture been resolved in sufficient detail to explain how it integrates diverse signals into a single phosphorylation event on eIF2α. GCN2 is composed of five major regions: an N‐terminal RWD (RING‐finger proteins, WD repeat‐containing proteins and the yeast DEAD‐like helicases) domain, a pseudokinase domain, an active kinase domain, a histidyl‐tRNA synthetase (HisRS)‐like domain, and a CTD (C‐terminal domain), each contributing a distinct layer of regulation [46, 48, 49] (Fig. 2).

A rich structural framework now underpins our understanding of these domains. High‐resolution crystal structures are available for the human kinase domain (PDB 7QQ6/7QWK/6N3L/6N3N/6N3O) [50, 51] and its yeast counterpart (PDB 1ZYC/1ZYD) [52]; for the RWD domain from human (PDB 7E2K/7E2M) [53], yeast (PDB 2YZ0) and mouse (PDB 1UKX) [54]; and for the CTD from yeast (PDB 4OTM) and mouse (PDB 4OTN) [55]. More recently, cryo‐EM structures have filled in the remaining gaps, capturing HisRS‐like domains from human GCN2 (PDB 8T7T), *Chaetomium thermophilum* (PDB 9CBS) and *Kluyveromyces marxianus* (PDB 9BF3), as well as a yeast Gcn2 dimer bound directly to the ribosomal 60S subunit (PDB 9F58) [56, 57, 58, 59]. Although a structure of the entire protein dimer is not yet available, advances in deep learning [60] allow us to generate plausible models that recapitulate features of the individual domain structures (Fig. 2).

Together, these structures reveal GCN2 as a stress sensor whose activation is orchestrated through a series of interlocking conformational restraints. The kinase domain adopts the canonical bilobed serine/threonine kinase fold, with the ATP‐binding pocket nestled in the hinge region (residues 801–806) [50, 52]. A large, highly charged serine/threonine‐rich ‘insert loop’ (residues 661–793) between strands β4 and β5 of the N‐lobe upstream of the activation loop is a unique feature of the eIF2α family of kinases [24, 61]. Although its function has not been explored fully for GCN2, in the related protein PERK, this loop becomes heavily phosphorylated on kinase activation and plays a role in substrate recruitment [61]. In the absence of stress, the HisRS‐like domain and CTD stabilise an inhibited dimeric state [58, 62, 63, 64]. Cryo‐EM studies now show that this autoinhibited arrangement is reinforced when GCN2 associates with the ribosomal 60S subunit, suggesting that GCN2 is held in a repressed configuration until ribosomal conditions change [58].

**Fig. 3:**
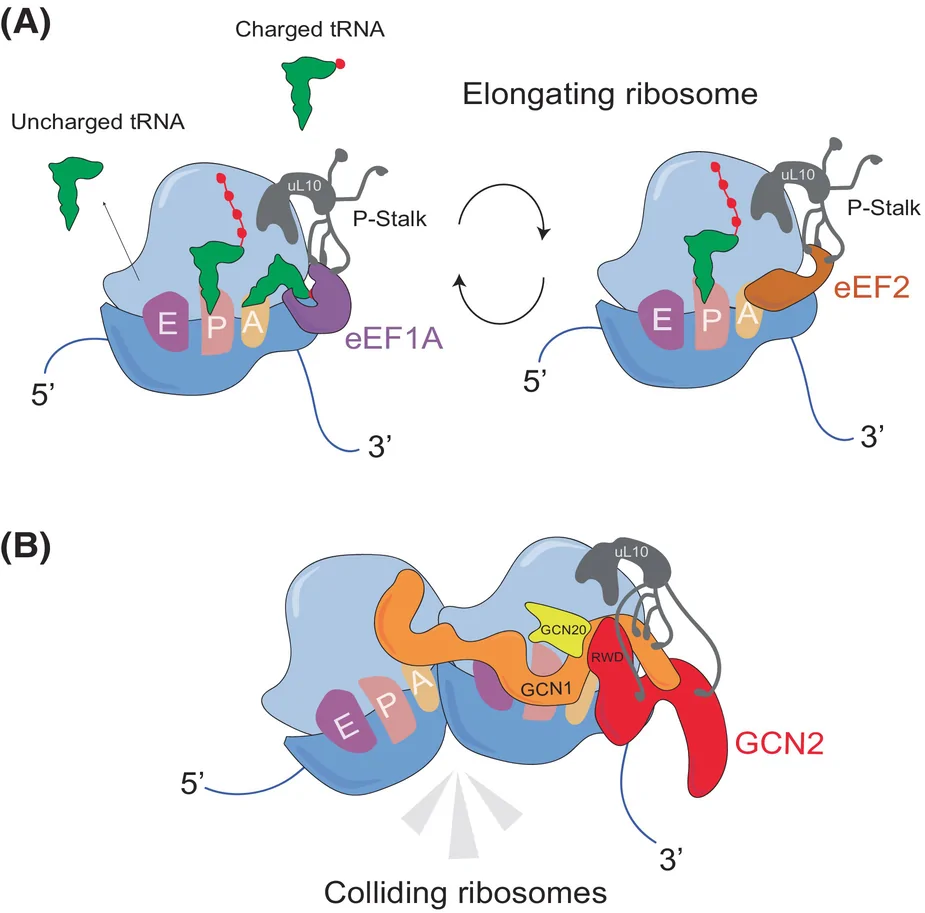
Detecting stalled ribosomes. (A) eEF1A delivers aminoacyl‐tRNA to the ribosome A‐site on the 69S ribosome subunit during polypeptide elongation. The P‐stalk binds GTP‐bound eEF1A, which sits adjacent to the P‐stalk base (uL10). Subsequently, eEF2 binds at the intersubunit space, bridging 40S and 60S, and interacts directly with the P‐stalk. This stimulates GTP hydrolysis, rotation of the 40S subunit, and movement of the peptidyl‐tRNA into the P site. (B) GCN1 binds to the leading ribosome in a collision stack. In yeast, Gcn20 stabilises GCN1 on the ribosome; in mammals, ABCF3 is the functional orthologue. Like the elongation factors in (A), the GCN complex has access to the A‐site and interacts with the P‐stalk. Threats to protein synthesis activate the kinase GCN2, initiating the integrated stress response (ISR). GCN2 is triggered by stalled ribosomes and uncharged tRNAs, which accumulate when amino acids are scarce. The ISR adjusts cellular physiology by promoting redox balance, protein quality control and mitochondrial optimisation. GCN2 and mTOR form a reciprocal, largely antagonistic signalling pair, coordinating growth and stress adaptation.

The pseudokinase domain, although catalytically inactive, is far from vestigial. It interacts with the active kinase domain to enhance eIF2α phosphorylation once GCN2 is released from its inhibited state [65]. Meanwhile, the RWD domain anchors GCN2 to GCN1, a key detector of stalled ribosomes, positioning the kinase precisely where uncharged tRNAs and ribosomal collisions accumulate [66, 67, 68, 69, 70, 71] (Fig. 3 and discussed later). The CTD further contributes to ribosome binding [64, 69, 72], completing a multidomain interface that couples GCN2 activation directly to translational stress.

Viewed together, these structural and biochemical insights depict GCN2 as a highly coordinated integrator of proteostatic cues. Its domains form a modular system that senses uncharged tRNAs, ribosome stalling and translational slowdown and then channels these signals into rapid eIF2α phosphorylation. This architecture explains how GCN2 can remain inert during normal translation yet respond decisively when amino acid availability or ribosome dynamics are perturbed.

#### Molecular basis of GCN2 stress sensing and activation

##### Uncharged tRNAs as activators of GCN2

Amino acid deprivation deprives the ribosome of the substrates required for peptide elongation, and under these conditions GCN2 is the principal ISR kinase that responds [27, 28]. Activation of the ISR leads to induction of ATF4 and CHOP, which in turn upregulate genes involved in amino acid transport and biosynthesis, including eleven aminoacyl‐tRNA synthetases that expand the pool of charged tRNAs [73] (Fig. 1). ISR signalling also enhances amino acid uptake through transporters such as CAT1, which imports cationic amino acids including arginine and lysine, thereby helping cells mitigate nutrient stress [73].

The earliest model of GCN2 activation focused on its ability to detect uncharged tRNAs. During amino acid starvation, uncharged tRNAs accumulate and bind directly to the HisRS‐like domain of GCN2 [48, 49, 64, 74, 75]. Mutational studies in yeast supported this model: disrupting residues required for tRNA binding impaired GCN2 activation [74, 75]. Structural work has since shown how tRNA binding destabilises inhibitory contacts between the kinase domain, the HisRS‐like domain, and the CTD, allowing GCN2 to transition from an autoinhibited dimer to an active configuration [50, 64, 76]. This rearrangement permits ATP binding and autophosphorylation of the activation loop, driving full kinase activation [50, 52]. Mutations that increase hinge flexibility (yeast R794G or F842L; human S808 or F855) mimic this conformational shift and render GCN2 constitutively active [52, 77].

#### Proteostatic and ribosome stress as triggers for GCN2 activation

It is now clear, however, that uncharged tRNA binding alone cannot account for the breadth of stresses that activate GCN2. The kinase also responds to perturbations in ribosome function itself. Ribosome stalling can be triggered by ultraviolet irradiation, oxidative stress or RNA alkylation, and each of these insults activates GCN2 [19, 78, 79, 80, 81, 82, 83, 84, 85]. When a ribosome stalls, trailing ribosomes may collide with it, generating a potent signal of translational distress [86, 87, 88]. Cells respond through two coordinated pathways: activation of the ISR via GCN2 and engagement of the Ribosome Quality Control (RQC) system. The RQC pathway, mediated by ZNF598 in mammals (Hel2 in yeast), ubiquitinates collided ribosomes to promote their disassembly and the degradation of incomplete nascent chains and defective mRNAs [89, 90, 91, 92, 93]. These pathways are mutually tuned: RQC activation suppresses GCN2, and loss of one pathway leads to hyperactivation of the other [94, 95]. In yeast, RQC appears to act at lower levels of stalling, preventing premature Gcn2 activation in the absence of significant stress [94].

GCN2's ability to sense ribosomal stress is rooted in its physical association with the 60S subunit, a property recognised since the 1990s [69]. GCN2 binds reversibly to 60S subunits under basal conditions and becomes activated when ribosomal function is perturbed [58, 68, 76, 84, 85]. A key structural feature of the 60S subunit is the P‐stalk, composed of two P1–P2 heterodimers and the uL10 protein, which together form a flexible arm that extends into the cytosol [96, 97, 98, 99, 100, 101]. GCN2 binds the P‐stalk with higher affinity than uncharged tRNAs [84]. uL10 engages inactive GCN2, while the C‐terminal tails of P1 and P2 stimulate its activation [83, 84] (Fig. 3). A recent cryo‐EM structure of the yeast Gcn2–60S complex revealed that Gcn2 occupies a site overlapping with the binding regions of elongation factors eEF1A and eEF2 [58]. This suggests a model in which, under normal conditions, cycling of charged tRNAs and elongation factors through the GTPase centre sterically limits GCN2–uL10 interactions [83, 84, 85]. When charged tRNAs become limiting, ribosomes stall, elongation factor cycling slows, and the P‐stalk becomes free to activate GCN2 [83, 84, 85].

GCN2 also engages multiple rRNA elements [58]. The CTD contacts helix H38, which interacts with A‐site tRNA and helix H84 in the central protuberance. The HisRS‐like domain binds helix H43 within the GTPase‐activating centre, while the pseudokinase domain interacts with helix H70, which stabilises large–small subunit association.

*In vitro* reconstitution experiments have further clarified the hierarchy of signals. Efficient GCN2 activation requires recruitment to stalled ribosomes by the disome sensor GCN1 [102] (Fig. 3). In this system, uncharged tRNAs are not strictly required for GCN2 to respond to amino acid stress, but they enhance activation when their anticodon matches the empty A‐site codon of the lead stalled ribosome [102]. GCN1 interacts with both the RWD domain of GCN2 and with a pair of collided ribosomes [66, 67, 68, 69, 70, 71]. In yeast, Gcn20 (the mammalian orthologue is ABCF3) forms a stable complex with Gcn1 to support Gcn2 activation [68, 71]. GCN1 shares homology with the N‐terminal region of the elongation factor eEF3, while GCN20 resembles its C‐terminal portion [71, 103]. eEF3, which is absent in mammals, promotes release of deacylated tRNA from the E‐site during elongation [104, 105]. By analogy, the GCN1–GCN20 complex may act to retain, rather than release, tRNA at the ribosome, facilitating transfer of uncharged tRNA from the A‐site to GCN2 [68, 70, 76].

### GCN2 and protein quality control pathways

#### GCN2–mTORC1 interplay under amino acid starvation stress

Amino acid availability is monitored by both GCN2 and mTORC1, but these pathways respond in opposite directions. Whereas GCN2 is activated by amino acid scarcity, mTORC1 is repressed under the same conditions [106]. Strikingly, leucine or arginine deprivation fails to inhibit mTORC1 in GCN2‐deficient cells, indicating that GCN2 contributes to mTORC1 repression during nutrient stress [107]. Mechanistically, GCN2 suppresses mTORC1 via Rag GTPases, and ATF4 reinforces this inhibition by inducing Ddit4 and Sestrin2 [108]. Ddit4 negatively regulates mTOR signalling, while Sestrin2 prevents mTORC1 recruitment to lysosomes, sustaining its inactivation [106]. In addition, GCN2‐dependent phosphorylation of FBXO22 promotes ubiquitination and inhibition of mTOR during amino acid deprivation [109].

The relationship between these pathways is bidirectional. mTORC1 integrates growth factor signalling as well as nutrient cues [110, 111, 112], and when forced to remain active during amino acid starvation, it can phosphorylate GCN2 to amplify ISR signalling [113]. In this setting, mTOR inhibition blocks ATF4 induction. Yet paradoxically, pharmacological inhibition of mTORC1 has also been reported to activate GCN2 via protein phosphatase 6, which forms a complex with GCN2 to promote its activation [114]. These findings underscore a context‐dependent interplay between mTORC1 and GCN2, reflecting the need for coordinated control of translation, metabolism and stress adaptation.

#### GCN2–autophagy axis in protein degradation

GCN2 contributes to proteostasis not only by regulating protein synthesis but also by modulating protein degradation pathways. Amino acid deprivation induces macro‐autophagy (hereafter autophagy) through several mechanisms, including at least one that requires GCN2 [115]. Autophagy sequesters cytoplasmic components into double‐membrane autophagosomes that fuse with lysosomes for degradation [116]. Through ATF4, GCN2 induces autophagy‐related genes such as MAP1LC3B and ATG5, thereby promoting autophagosome formation [117, 118, 119, 120]. Rapid autophagy induction can also occur downstream of GCN2 and eIF2α phosphorylation independently of ATF4: as little as 1 h of leucine deprivation triggers autophagy in fibroblasts and mouse liver in a GCN2‐dependent, ATF4‐independent manner [121].

This pathway has important implications for inflammatory disease. IDO1, an enzyme expressed in monocytic, epithelial and endothelial cells, depletes tryptophan during inflammation and thereby activates GCN2 [122, 123]. The resulting induction of autophagy influences cell survival and tissue protection. In a mouse model of anti‐glomerular basement membrane kidney disease, the IDO1–GCN2 axis limited inflammatory injury by promoting autophagy in podocytes [124]. In the gut, GCN2 activation in antigen‐presenting and epithelial cells suppressed inflammation by inducing autophagy to reduce mitochondrial reactive oxygen species [120]. These findings position GCN2 as a key regulator linking nutrient stress to autophagic control of inflammation.

#### GCN2 and the ubiquitin–proteasome system

Proteostasis also relies on the ubiquitin–proteasome system (UPS), which targets proteins for degradation via polyubiquitylation. In mammals, ubiquitin is encoded by four genes, with *UBB* and *UBC* providing the polyubiquitin chains used for proteasomal targeting [125, 126]. NRF1 and NRF2 upregulate UBC expression during stress [127, 128], and both transcription factors are induced by the ISR through ATF4, establishing a regulatory axis linking GCN2 signalling to the UPS [129, 130]. Both ATF4 and UBC are required to maintain tRNA‐Gln charging during amino acid starvation [131].

When the UPS becomes impaired or overloaded, cells form stress granules to temporarily sequester ubiquitylated and misfolded proteins [132, 133, 134]. These membraneless organelles arise through liquid–liquid phase separation [135]. During UPS inhibition, eIF2α phosphorylation is primarily mediated by GCN2 [136, 137]. Phosphorylation of eIF2α suppresses translation initiation, leaving untranslated mRNAs, stalled pre‐initiation complexes, and RNA‐binding proteins available to nucleate stress granules [138, 139, 140]. RNA‐binding proteins such as TIA1, TIAR, G3BP1 and PABP recognise these untranslated mRNAs and, through their low‐complexity domains, drive phase separation and granule assembly [139, 141, 142]. In this way, GCN2 contributes to proteostasis not only by modulating translation but also by shaping the cellular response to proteotoxic stress.

#### Organelle proteostasis: the role of GCN2 in mitochondrial stress responses

Mitochondria are highly dynamic organelles that continuously remodel their network through fusion and fission, adjusting their morphology to match cellular metabolic demands [143]. Excessive mitochondrial fragmentation, however, is increasingly recognised as a hallmark of mitochondrial dysfunction and is implicated in a wide range of neurodegenerative and chronic inflammatory diseases, and in ageing [144, 145, 146, 147, 148, 149, 150, 151]. The ISR contributes to mitochondrial homeostasis by coordinating both transcriptional and translational responses that support organelle integrity.

ATF4 plays a central role in this process by inducing mitochondrial chaperones such as HSPA9 and proteases including LONP1, thereby reinforcing mitochondrial protein quality control [152]. Perturbations in mitochondrial respiration can also feed directly into GCN2 signalling. Inhibition of mitochondrial complex I activates GCN2‐dependent ISR signalling in myoblasts, an effect that can be reversed with the GCN2 inhibitor GCN2iB [152]. Conversely, pharmacological activation of GCN2 by halofuginone has been shown to mitigate mitochondrial dysfunction by promoting adaptive mitochondrial elongation, enhancing mitochondrial respiration and preventing calcium ionophore–induced fragmentation [153].

Together, these findings position GCN2 as an important regulator of mitochondrial proteostasis. By integrating signals from nutrient stress, translational slowdown and mitochondrial dysfunction, GCN2 helps maintain mitochondrial morphology and function, linking cytosolic stress sensing to organelle‐level adaptation.

### Dysregulated GCN2 signalling and proteostasis in disease

#### Pulmonary hypertension

Pulmonary arterial hypertension (PAH) occurs with mean pulmonary arterial pressure, measured by right heart catheterisation, exceeding 20 mmHg at rest, which imposes an unphysiological load on the right ventricle leading to heart failure [154, 155]. Familial PAH is most often linked to heterozygous mutations in *BMPR2*, but mutations in GCN2 have been described [156, 157, 158]. More than 50 potentially pathogenic alleles of *EIF2AK4*, which encodes GCN2, have been identified in PAH patients [159, 160, 161, 162]. In rare, fatal subtypes of PAH such as pulmonary veno‐occlusive disease (PVOD) and pulmonary capillary haemangiomatosis (PCH), biallelic loss‐of‐function mutations in GCN2 disrupt proteostasis by abolishing stress‐responsive translational control, leading to uncontrolled vascular cell proliferation and poor survival [159, 161, 163]. This dysregulation of proteostasis reduces BMP‐dependent SMAD1/5/8 phosphorylation in pulmonary endothelial cells, enhancing abnormal proliferation [164]. Downstream ISR effectors such as CHOP and HO‐1 are aberrantly activated in PVOD, reflecting maladaptive proteostasis signalling [164]. Additional pathways, including ATF3–p38 MAPK signalling and PI3K–AKT/mTOR activation, further link GCN2 loss to altered collagen synthesis and vascular remodelling [165, 166, 167]. Chemotherapeutic agents such as mitomycin‐C (MMC) exacerbate this phenotype by depleting pulmonary GCN2 [168]. It is noteworthy that such alkylating agents modify ribosomes and mRNA, which is likely to induce ribosomal stress [169, 170].

#### Cancer

Transient activation of the ISR can support tumour cell survival by stabilising proteostasis, whereas prolonged signalling drives apoptosis through CHOP‐mediated death receptor pathways and PPP1R15A induction [38, 171]. Large‐scale multiomics analyses indicate that approximately 13% of cancer cell lines depend on GCN2 for viability, far more than on any other eIF2α kinase, highlighting its particular importance in malignant settings [172]. In some cases, cells surviving chemotherapy‐induced proteotoxic stress acquire a selective dependency on GCN2, likely through an increased requirement for ISR‐induced amino acid sufficiency and optimised redox homeostasis. Elevated GCN2 activity additionally enables a broad range of tumour cells to withstand nutrient stress and maintain proteostasis, supporting growth across multiple cancer types including colon, lung, breast, prostate and liver cancers [115, 173, 174, 175]. By regulating redox balance and mitophagy, GCN2 also contributes to chemoresistance [176, 177].

Pharmacological inhibition of GCN2 is therefore being explored as a therapeutic strategy. Small molecule inhibitors such as GCN2iB and APL4098 reduce proliferation and induce apoptosis in cancer models [178, 179, 180, 181]. In prostate and hepatocellular carcinoma, GCN2 promotes expression of amino acid transporters, enabling tumour cells to sustain proteostasis under nutrient limitation; inhibition of GCN2 suppresses tumour growth in these contexts [182, 183]. In colon adenocarcinoma, GCN2 enhances ribosome biogenesis even under nutrient‐replete conditions, directly linking translational capacity to tumour progression [184].

But the relationship between GCN2 and cancer is not strictly linear. Excessive or prolonged GCN2 activation can overwhelm proteostasis, triggering apoptosis and senescence [183, 185]. As a result, both inhibition and paradoxical activation of GCN2 are being pursued therapeutically. Several GCN2 activators, including NXP800 and HC7366, have now entered clinical testing [186, 187, 188], reflecting growing interest in manipulating this pathway to exploit tumour vulnerabilities. Mechanistically, such molecules bind to the ATP‐binding pocket of the kinase domain in its active conformation and thereby propagate a conformational change that activates the second GCN2 protomer within the dimer [189, 190, 191, 192].

#### Inflammation and immune diseases

GCN2 also plays a central role in immune cell proteostasis. By sensing amino acid deprivation, it modulates T‐cell and dendritic‐cell function, aligning protein synthesis with immune activation and metabolic capacity [80, 193, 194, 195]. In the intestine, GCN2 suppresses inflammasome activation and limits Th17 differentiation during amino acid starvation, preventing proteostatic overload and excessive inflammation [120, 196]. Yet GCN2's influence is context‐dependent: In airway inflammation, it promotes Th9 differentiation, demonstrating that its effects can be either protective or pathogenic depending on the immunological environment [195].

The IDO1‐GCN2 axis is particularly relevant in immune regulation. IDO1 depletes tryptophan during inflammation, and GCN2 functions as a molecular sensor that allows T cells to detect and respond to this metabolic shift [80]. IDO‐expressing plasmacytoid dendritic cells activate the GCN2 pathway in responding T cells, inducing profound anergy, a response that is abolished in GCN2‐deficient T cells [80]. Through this mechanism, GCN2 links amino acid metabolism to immune tolerance and inflammatory control.

## Discussion

GCN2 has emerged as a central integrator of nutrient stress, translational control and proteostasis. It is far more than a simple amino acid sensor, but rather acts as a multifaceted coordinator of cellular homeostasis. This versatility helps explain why dysregulated GCN2 signalling contributes to such a wide spectrum of diseases, including pulmonary hypertension, cancer and inflammatory disorders.

Despite major advances in structural biology, several aspects of GCN2 activation remain incompletely understood. Cryo‐EM studies have clarified how GCN2 engages the ribosome, yet the precise sequence of events that transitions the kinase from autoinhibited to fully active is still being resolved. The relative contributions of uncharged tRNA binding, P‐stalk engagement and GCN1‐mediated recruitment likely vary across stress contexts, while the hierarchy and integration of these signals remain open questions. Similarly, the extent to which GCN2 activation is spatially regulated—whether at specific ribosomal subpopulations, organelle‐associated translation sites or stress granules—has not been fully explored.

Our structural understanding of the activation of GCN2 by therapeutic small molecules remains incomplete. Type 1 kinase inhibitors bind to the active conformation, where key structural elements in the catalytic core are positioned to support ATP binding and phosphate transfer (activation loop DFG‐Asp‐in, αC‐helix‐in conformation) [197]. Type 2 inhibitors bind the inactive conformation, in which these elements adopt an outward‐facing arrangement (DFG‐out, αC‐helix‐out) [197]. Type 1.5 inhibitors recognise an intermediate state, where the activation loop resembles the active conformation, but the αC‐helix has shifted outward. Several type 1.5 inhibitors exhibit a biphasic effect, activating GCN2 at low concentrations but inhibiting it at higher concentrations [191, 198, 199]. GCN2iB, for example, is a relatively selective type 1.5 inhibitor that activates GCN2 at sub‐micromolar doses [162, 191]. The mechanism for this has not yet been proved, but one proposed mechanism is that drug‐induced stabilisation of one subunit of the GCN2 dimer in an active conformation is transmitted to the partner subunit, promoting activation of the dimer [199, 200]. The requirement for GCN2 binding partners in such drug‐induced activation has also not been fully resolved, although most small molecules appear to require GCN1 to activate the kinase, suggesting they may function by exaggerating basal activation [189].

Another emerging area concerns the interplay between GCN2 and other proteostasis regulators. The reciprocal regulation between GCN2 and mTORC1 is clearly more complex than a simple nutrient‐sensing toggle, with evidence for both inhibitory and activating crosstalk depending on cellular context. How these pathways coordinate translational reprogramming, autophagy induction and metabolic adaptation remains unresolved. Likewise, the relationship between GCN2 and the ubiquitin–proteasome system, particularly in the context of stress granule dynamics, warrants deeper mechanistic investigation. Moreover, evidence points to the existence of ISR‐independent functions for GCN2 in regulating ribosome biogenesis, which could have profound consequences for proteostasis [184, 201]. These ISR‐independent mechanisms require further elucidation.

The role of GCN2 in organelle proteostasis, especially mitochondrial quality control, is only beginning to be appreciated. Whether GCN2 directly senses mitochondrial dysfunction or responds indirectly through translational slowdown and metabolic cues is not yet clear. Given the centrality of mitochondrial stress in neurodegeneration, metabolic disease and ageing, dissecting this axis may reveal new therapeutic opportunities.

Disease‐focused studies also highlight unresolved questions. In pulmonary hypertension, it remains unclear why loss‐of‐function mutations in *EIF2AK4* drive such a specific vascular phenotype, and how impaired ISR signalling intersects with BMP pathway dysfunction. In cancer, the dual nature of GCN2 as both a survival factor under nutrient stress and a potential driver of apoptosis when hyperactivated raises the possibility of context‐dependent therapeutic strategies. Understanding which tumours rely on GCN2 for proteostasis, and which are vulnerable to its overactivation, will be essential for rational drug development. Similarly, in immune regulation, the divergent roles of GCN2 in promoting tolerance versus inflammation underscore the need to map cell‐type‐specific and microenvironment‐specific signalling logic.

## Conclusion

Taken together, the field now stands at a point where the molecular architecture and broad physiological roles of GCN2 are well defined, but the systems‐level logic that governs its activation and downstream consequences remains incompletely mapped. As our understanding deepens, GCN2 is likely to shift from being viewed as a classical nutrient stress kinase to a broader guardian of proteome integrity. Clarifying how this single enzyme integrates metabolic, translational and organelle‐derived signals will be key to unlocking new therapeutic strategies for diseases driven by proteostatic imbalance.

## Conflict of interest

The authors declare no conflict of interest.

## Author contributions

JZ and SJM conceived and wrote this review and designed the illustrations.
